# Brain State-Dependent Non-Invasive Neurostimulation with EEG Feedback: Achievements and Prospects (Review)

**DOI:** 10.17691/stm2023.15.5.04

**Published:** 2023-10-30

**Authors:** A.I. Fedotchev, A.A. Zemlyanaya

**Affiliations:** DSc, Leading Researcher, Laboratory of Biosystems Regulating Mechanisms; Institute of Cell Biophysics of the Russian Academy of Sciences, 3 Institutskaya St., Pushchino, Moscow Region, 142290, Russia; MD, PhD, Senior Researcher, Department of Exogenic and Organic Disorders and Epilepsy; Moscow Research Institute of Psychiatry — Branch of the Serbsky State Scientific Center for Psychiatry and Narcology of the Ministry of Health of Russia, Bldg. 10, 3 Poteshnaya St., Moscow, 107076, Russia

**Keywords:** non-invasive brain stimulation, electroencephalogram, EEG, feedback, brain microstates, EEG-controlled adaptive neurostimulation, cognitive rehabilitation, functional state correction

## Abstract

Non-invasive brain stimulation with electroencephalogram (EEG) feedback is an intensively developing and promising area of neurophysiology. The review considers the literature data over the past 5 years on the achievements and promising directions for the further development of this research line. Modern data on the developed approaches to the practical use of various types of brain state-dependent adaptive neurostimulation with EEG feedback were analyzed. The main attention is paid to the studies using non-invasive magnetic and electrical stimulation, as well as acoustic and audiovisual stimulation. The paper considers the possibilities and prospects for using these technologies in clinical medicine. The results of the authors' own research are presented.

## Introduction

The development and clinical application of non-invasive brain stimulation methods is a promising and intensively advancing field of neurophysiology, which is called “non-invasive brain stimulation” (NIBS). Transcranial magnetic stimulation and transcranial direct and alternating current stimulations are considered to be the most developed NIBS techniques [1-3], so are rhythmic sensory stimulation (acoustic, video- and audiovisual) [4-6]. NIBS techniques enable to achieve improved outcomes in neurological rehabilitation of neurologic patients [7-12], in cognitive and stress-induced impairments [13-18], when treating psychiatric disorders [19-24], and in enhancing cognitive functions in healthy people [25-30].

Despite the intensive development and researchers’ increased interest, existing NIBS techniques have a number of drawbacks, such as low efficiency, high variability, and poor reproducibility [31-33]. The reason is that standard NIBS techniques do not reckon with the dynamic nature of the neural endogenous oscillatory activity, and the stimuli are delivered during different physiological brain microstates leading to the high variability of a single-stimulus effect and a weak cooperative stimulation effect [34-36].

To eliminate the shortcomings, some authors recommend using closed-loop brain state-dependent neurostimulation protocols, which take into consideration the ongoing brain microstates dynamics [37-40]. The real-time electroencephalogram (EEG) data is an optimal source of the feedback signals [41-44] due to EEG advantages, such as non-invasiveness, high temporal resolution, ease of use, and real-time data extraction [45-47]. The EEG-controlled stimulation protocols enable NIBS techniques to achieve a highly personalized effect and offer physiologically informed adaptive neuromodulation [48-52].

Over the past 5 years, the number of studies on the effects of the closed-loop brain state-dependent non-invasive neurostimulation has increased exponentially. The plethora of recent publications and a wide variety of specific experimental approaches necessitate the literature data summary on the achievements and promising trends for further NIBS development. Therefore, this review studies the present-day data on the developed approaches to the practical use of various types of the closed-loop brain state-dependent adaptive neurostimulation. The article focuses on non-invasive magnetic and electric influences, as well as acoustic and audiovisual stimulation. The authors present the possibilities and prospects of the clinical application of the techniques. The review shows the results of the authors’ own research. The literature search was carried out on the PubMed/ MEDLINE database using the key words “closed-loop stimulation” and “adaptive neurostimulation”.

## Brain state-dependent non-invasive neurostimulation achievements

The advantages of the EEG feedback when correcting many unfavorable functional states have been demonstrated in a number of studies. The widespread implementation of the EEG-based closed-loop neurofeedback into the previously used methods of human sleep regulation is one of the most intensively developing research fields. Its conceptual basis is based on the theoretical and methodological ideas that non-invasive sensory closed-loop stimulation can improve sleep quality, enhance cognitive functions and memory consolidation [53-55]. These effects have been demonstrated in the experiments with different sensory closed-loop stimulations such as transcranial electrical stimulation [56-59], transcranial magnetic stimulation [60-62], and acoustic stimulation [63-65]. Success was achieved with different EEG feedback parameters: the EEG phase-specific rhythm [66, 67], the occipital alpha rhythm power [68, 69], slow-wave EEG components [70, 71] and EEG sleep spindles [72].

The EEG-controlled acoustic stimulation is effective in other clinical applications. The acoustic stimuli, which are translated real-time from dominant EEG frequencies by software, lead to a clinically significant decrease in the post-traumatic stress symptoms [73]. According to the authors [74], the online update of patients-own EEG patterns and resonance between the audible tones and oscillating brain networks allow the brain to auto-calibrate, relax, and overcome persisting pathological states.

Another variant of the EEG-controlled acoustic stimulation has been successfully used in bioacoustic correction consisting in presenting to a person the acoustic computer-transformed signals obtained during an ongoing EEG. The method enables to “listen to” the brain real-time work and correct unfavorable functional organism states in cognitive and emotional-volitional disorders [75, 76].

The “Music of the brain” concept, according to which EEG parameters transformation increases the effectiveness of musical therapeutic effects, is the basis of our original research [77]. There was developed a version of the closed-loop acoustic stimulation in the form of classical music, the volume of which is automatically modulated by the ongoing amplitude of the dominant spectral peak in the range of the EEG alpha rhythm, or EEG alpha oscillator [78, 79]. The method was supplemented by the computer conversion of the ongoing EEG alpha oscillator amplitude into music-like signals resembling flute sounds in timbre and smoothly varying in pitch and intensity [80]. The developed musical neurointerfaces have been successfully tested to correct many functional disorders, as well as to eliminate the risks of specialist reliability [81] and in the cognitive rehabilitation in the elderly [82].

We have then shown that photostimulation, which is automatically generated in real time based on digitized values of the ongoing EEG, also has positive effects [83]. The combination of the described approaches resulted in the development of the audiovisual adaptive neurostimulation with double feedback from the human EEG [84]. The method consists in simultaneous stimulation with music-like stimuli generated based on the current amplitude of the alpha EEG oscillator and with rhythmic light stimulation generated based on the current EEG. The method advantages are the high personalization and therapeutic effectiveness due to the feedback from person’s own bioelectric characteristics, the involvement of mechanisms of multisensory integration, neuroplasticity and resonance brain mechanisms in the functional state normalization under stimulation; other advantages include automatic, without conscious patient efforts, management of therapeutic sensory effects, which makes it possible to use adaptive neurostimulation to correct adverse state shifts in patients with altered levels of consciousness, the elderly, and children [85, 86].

These advantages enabled EEG-controlled light-sound neurostimulation to be successful in stress-induced states correction [87, 88], the functional state optimization [89], and correction of its adverse shifts [90, 91], in cognitive rehabilitation of high-tech specialists [92], as well as in clinical studies in cognitive rehabilitation in stroke patients [93] and in the treatment of post-traumatic stress and professional burnout [94].

Thus, there is a wide range of conditions when brain-dependent EEG-controlled neurostimulation can be successfully used, as well as the specific the Table). The number of studies on the subject is characteristics of the therapeutic stimulations (see increasing annually, which indicates it to be promising.

**Table:** Dynamics of non-invasive EEG-controlled methods development

Study aim/effect	Stimulation type	EEG control parameter	Reference
Sleep quality improvement, memory consolidation	Transcranial electric stimulation	Slow-wave EEG components	Ketz et al., 2018 [56]
Correction of stress-induced conditions	Classical music	Alpha EEG oscillators	Fedotchev, 2018 [78]
Elimination of stress-induced risks of specialist reliability	Classical music	Alpha EEG oscillators	Fedotchev et al., 2018 [79]
Post-traumatic stress treatment	Acoustic stimuli	Dominant EEG rhythms	Shaltout et al., 2018 [73]
Correction of functional disorders	Sound/music-like stimulation	Alpha EEG oscillators	Zemlyanaya et al., 2018 [80]
Sleep quality improvement, cognitive control enhancement	Transcranial electric stimulation	EEG rhythm phase	Mansouri et al., 2018 [66], 2019 [57]
Sleep quality improvement, memory consolidation	Acoustic stimuli	Sleep spindles in EEG	Ngo et al., 2019 [72]
Correction of stress-induced conditions	Rhythmic light stimulation	Digitized native EEG	Fedotchev, 2019 [83]
Correction of stress-induced conditions	EEG-controlled light-sound stimulation	Alpha EEG oscillators + native EEG	Fedotchev et al., 2019 [84]
Revelation of neurodegenerative disorders markers	Transcranial magnetic stimulation	Induced potentials	Poydasheva et al., 2019 [60]
Elimination of stress-induced risks of specialist reliability	Sound/music-like stimulation	Alpha EEG oscillators	Fedotchev et al., 2019 [81]
Treatment of posttraumatic stress disorder	Acoustic stimuli	Dominant EEG rhythms	Tegeler et al., 2020 [74]
Cognitive rehabilitation of elderly patients	Sound/music-like stimulation	Alpha EEG oscillators	Fedotchev et al., 2020 [82]
Enhancement of cognitive functions through neuroplasticity induction	Transcranial electric stimulation	EEG occipital alpha rhythm	Zarubin et al., 2020 [58]
Depressive disorder therapy	Transcranial magnetic stimulation	EEG alpha and theta rhythms	Zrenner et al., 2020 [68]
Correction of stress-induced conditions	Sound/music-like stimulation	Alpha EEG oscillators	Fedotchev, 2020 [87]
Sleep quality improvement, memory consolidation	Acoustic stimuli	Slow-wave EEG components	Schneider et al., 2020 [70]
Enhancement of cognitive and visual functions	Transcranial electric stimulation	EEG temporal alpha rhythm	Stecher et al., 2021 [69]
Human functional state optimization	EEG-controlled light-sound stimulation	Alpha EEG oscillators + native EEG	Fedotchev et al., 2021 [89]
Cognitive rehabilitation in stroke patients	EEG-controlled light-sound stimulation	Alpha EEG oscillators + native EEG	Mukhina et al., 2021 [93]
Enhancement and clarification of adaptive neurostimulation processes	Transcranial magnetic stimulation	Peak values phase of EEG rhythms	Shirinpour et al., 2021 [67]
Treatment of posttraumatic stress disorder and burnout	EEG-controlled light-sound stimulation	Alpha EEG oscillators + native EEG	Fedotchev et al., 2021 [94]
Bioacoustic correction of patients' condition	EEG-controlled acoustic stimulation	Frontal and occipital EEG	Ivanova, Kormushkina, 2021 [75]
Correction of negative functional conditions	EEG-controlled light-sound stimulation	Alpha EEG oscillators + native EEG	Fedotchev et al., 2021 [90]
Enhancement of effects in stimulation parameters optimization	Transcranial electric stimulation	Slow-wave EEG components frequency	Ladenbauer et al., 2022 [59]
Depressive disorder therapy	Transcranial magnetic stimulation	EEG prefrontal alpha rhythm	Faller et al., 2022 [61]
Sleep quality improvement, memory consolidation	Acoustic stimuli	Slow-wave EEG components	Debellemaniere et al., 2022 [64]
Post-COVID syndrome treatment	EEG-controlled light-sound stimulation	Alpha EEG oscillators + native EEG	Polevaya et al., 2022 [95]
Sleep quality improvement, memory consolidation	Acoustic stimuli	Slow-wave EEG components	Ngo, Staresina, 2022 [63]
Enhancement of effects in considering EEG phase	Transcranial magnetic stimulation	EEG occipital alpha rhythm phase	Ding et al., 2022 [62]
Bioacoustic correction of patients’ condition	EEG-controlled acoustic stimulation	Frontal and occipital EEG	Shchegolkov et al., 2022 [76]
Sleep quality improvement, memory consolidation	Acoustic stimuli	Slow-wave EEG components	Ruch et al., 2022 [71]
Cognitive rehabilitation of a specialist	EEG-controlled light-sound stimulation	Alpha EEG oscillators + native EEG	Fedotchev, 2022 [92]
Sleep quality improvement, activation of autonomous functions	Acoustic stimuli	Dominant EEG rhythms	Tegeler et al., 2023 [65]

## Brain-dependent non-invasive neurostimulation prospects

Intensive and successful development of brain-dependent non-invasive neurostimulation has determined numerous beliefs about the prospects of using the method. It is noted that by the year of 2035 the non-invasive neurotherapy will have been based on neuromodulation devices, which are already effective to treat motor disorders, epilepsy, pain, depression, and other neurological disorders, due to the progress in understanding neuroanatomic networks and mechanisms of the neurostimulation with feedback from highly specific biomarkers including personalized EEG characteristics [96]. By now, the attempts to find highly specific EEG biomarkers enabled to demonstrate the possibilities of many individual EEG characteristics, such as short (50-100 ms) stable resting EEG microstates [97], interictal spikes [98], and the slow EEG wave phase [99].

The researches aimed at improving brain stimulation algorithms with feedback are integral for considering the prospects for developing brain-dependent non-invasive neurostimulation. Thus, there has been developed a reliable adaptive neuromodulation algorithm able to thoroughly track the trajectories of current brain conditions for effective brain diseases treatment and brain functions improvement [100]. A manual on electrophysiological registration and brain stimulation has been published, which allows the user to master the EEG data analysis and adjust immediately the stimulation parameters in feedback protocols [101]. Since the natural frequencies of neural activity can serve as precise targets of rhythmic stimulation effects [102-104], the methodology of optimal EEG preprocessing to increase the effectiveness of EEG-controlled neurostimulation seems promising [105].

The issue of the EEG-controlled adaptive neurostimulation development has been considered in our recent studies [84, 106]. Since EEG-controlled adaptive neurostimulation is based on the automatic modulation of sensory stimuli by the person’s own EEG rhythm components, one of the possible ways to increase its effectiveness might be a prior amplification of the modulating factor, i.e. the subject’s EEG. A resonance scanning technique is used for this purpose consisting in LED photostimulation with a step-by-step increasing frequency in the range of theta, alpha, and beta EEG rhythms [107].

Resonance scanning prior to adaptive neurostimulation significantly has been shown to increase the effectiveness of EEG-controlled adaptive neurostimulation in the treatment of post-COVID syndrome [95] and eliminating the consequences of exam stress in university students [108]. The resonance scanning combined with EEG-controlled adaptive neurostimulation has shown an increase in the alpha EEG rhythm power accompanied by a decrease in stress levels, an improved emotional state and cognitive performance due to the progressive involvement of resonant and integration brain and neuroplasticity. It is concluded that the developed combined approach to neurostimulation can be used after additional experimental studies in various rehabilitation measures, in the correction and rehabilitation of the extreme profession specialists’ state, in educational institutions to enhance human cognitive activity and learning processes.

## Conclusion

Brain-dependent non-invasive neurostimulation with EEG feedback is an intensively developing and promising neurophysiology field. The closed-loop brain stimulation enables to achieve high personalization and the effectiveness of therapeutic effects by taking into consideration the dynamics of the brain microstates.

The automatic modulation of sensory stimuli by the current EEG parameters is considered to be a promising research topic. Automatic control of therapeutic sensory stimuli makes it possible to use EEG-controlled adaptive neurostimulation to correct adverse state shifts in patients with altered levels of consciousness, the elderly, and children. The use of preliminary resonance scanning is especially promising; it causes the activation of potential EEG resonators and increases the brain reactivity to subsequent EEG-controlled adaptive neurostimulation. As a result of a combination of exogenous and endogenous rhythmic stimulation, positive psychophysiological effects are recorded after a single therapeutic effect. Such a combined approach to neurostimulation can be used in a wide range of rehabilitation procedures.
